# Association of a modified body shape index with cognitive impairment in middle-aged and elderly Chinese adults: a cross-sectional analysis from CHARLS

**DOI:** 10.3389/fnut.2025.1589898

**Published:** 2025-06-04

**Authors:** Guotao Liu, Qiong Xu, Suhua Zhang, Jianyuan Zhang

**Affiliations:** ^1^Department of Health Care, Qilu Hospital (Qingdao), Cheeloo College of Medicine, Shandong University, Qingdao, Shandong, China; ^2^Department of Neurology, Qilu Hospital (Qingdao), Cheeloo College of Medicine, Shandong University, Qingdao, Shandong, China; ^3^Qingdao Clinical Research Center for Rare Diseases of Nervous System, Qingdao, China

**Keywords:** A Body Shape Index, ABSI, cognitive impairment, CHARLS, cross-sectional study

## Abstract

**Purpose:**

This study aimed to investigate the association between A Body Shape Index (ABSI) and cognitive impairment in middle-aged and elderly Chinese adults.

**Patients and methods:**

We analyzed baseline data from the 2011 China Health and Retirement Longitudinal Study (CHARLS), including 6,762 participants aged ≥45 years. The modified ABSI of adults in China was calculated using waist circumference (WC), body mass index (BMI), and height. Cognitive function was assessed through episodic memory and cognitive status tests, with impairment defined as a composite score ≤11. Multivariate logistic regression, fitted smoothing curves, and subgroup analysis were employed to evaluate the associations and interactions. Receiver operating characteristic (ROC) curves were used to compare the diagnostic performance of ABSI, BMI, and WC.

**Results:**

After full adjustment, each 0.01-unit ABSI increase conferred a 45.7% higher risk of cognitive impairment (odds ratio [OR] = 1.457). Participants in the highest ABSI quartile had a 48.3% higher risk compared to the lowest quartile (*p* for trend < 0.001). Dose–response analysis revealed a positive relationship (*p* < 0.001) between ABSI and cognitive impairment. ABSI demonstrated superior diagnostic accuracy (Area Under the Curve [AUC] = 0.603) compared to BMI (AUC = 0.570) and WC (AUC = 0.548).

**Conclusion:**

Elevated ABSI independently predicts cognitive impairment in Chinese middle-aged and older adults. ABSI demonstrates better diagnostic accuracy compared to BMI and WC. These findings highlight ABSI’s utility as a cost-effective anthropometric tool for cognitive risk stratification in aging populations.

## Introduction

Cognitive impairment, encompassing conditions from mild cognitive impairment (MCI) to dementia, represents a significant public health challenge, particularly in aging populations (1). A 2020 epidemiological study revealed that China alone had 38.77 million individuals aged 60 years or older diagnosed with MCI and an additional 15.07 million suffering from dementia (2). The substantial socioeconomic impact of these cognitive disorders on global healthcare systems underscores the critical need for early detection and intervention strategies targeting modifiable risk factors associated with cognitive decline.

Obesity significantly contributes to the global burden of disease, primarily leading to cardiovascular diseases, type 2 diabetes, and various cancers, resulting in a decline in average human life expectancy. By 2050, the global number of overweight and obese adults will reach 3.8 billion, with China accounting for 627 million (3). This poses serious challenges to both medical management and societal prevention efforts. For a long time, body mass index (BMI) has been widely used as the standard definition for overweight and obesity in clinical practice and research. However, there has been ongoing debate regarding its limitations, such as low sensitivity, inability to distinguish between fat mass and muscle mass, and failure to assess abdominal obesity (4). While waist circumference (WC) can reflect visceral fat accumulation associated with abdominal obesity, it does not account for the influence of height. Therefore, Krakauer et al. developed a new obesity index, the A Body Shape Index (ABSI), by standardizing WC with BMI and height based on the body shape characteristics of the U. S. population (5). Building on this, in 2020, Wang et al. constructed a modified ABSI tailored to the body shape characteristics of the Chinese population, making it more suitable for Chinese adults (6).

Emerging evidence has established significant associations between the ABSI and various health outcomes, including arterial stiffness (7), cardiovascular events (8), psychological disorders (9), diabetes (10), bone mineral density (11), cancer (12), and all-cause mortality (13). A 2024 study further identified a correlation between elevated ABSI levels and cognitive decline in the U.S. population aged 60 years and older (14). Despite the development of modified ABSI formula tailored for Chinese adults by Wang et al. (6), the relationship between modified ABSI and cognitive function remains unexplored in large-scale Chinese populations. This study hypothesized that higher ABSI levels may raise the risk of cognitive impairment and aimed to explore this link systematically, while also assessing potential moderating factors that could affect the relationship in Chinese populations.

## Materials and methods

### Study population

This investigation utilized data from the China Health and Retirement Longitudinal Study (CHARLS), a nationally representative survey hosted by Peking University.^1^ CHARLS gathers detailed longitudinal data on individuals aged 45 years or older in China, with a specific emphasis on health conditions within aging populations. The initial survey wave (2011) covered 17,708 respondents from 450 rural and urban communities distributed across 150 administrative regions, offering a comprehensive profile of middle-aged and elderly demographics (15). Data collection employed computer-assisted personal interviewing (CAPI) methodology, with biennial follow-ups conducted to update information on demographics, health behaviors, social engagement, and insurance coverage. Ethical approval for this study was granted by the Institutional Review Board of Peking University (IRB00001052-11015), and all protocols complied with the Declaration of Helsinki. Written informed consent was obtained from each participant prior to enrollment.

**Figure 1:**
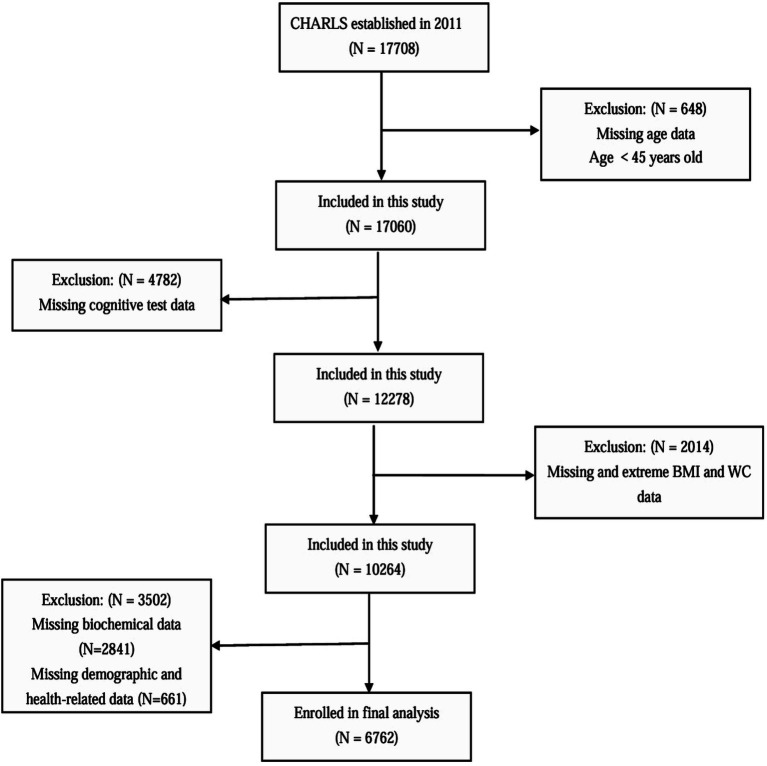
Participant selection process flowchart.

For this cross-sectional analysis, baseline data from the 2011 CHARLS cohort were analyzed. Participants meeting any of the following exclusion criteria were omitted: age below 45 years, incomplete and extreme anthropometric measurements (BMI/WC), missing cognitive evaluations, insufficient biochemical data, or incomplete demographic/health records. After applying these criteria, the final analytical sample comprised 6,762 eligible participants (Figure 1).

### Cognitive function assessment

Cognitive performance in the CHARLS cohort was assessed using protocols adapted from the Health and Retirement Study (HRS) (16). The evaluation framework comprised two core components: cognitive status and episodic memory capacity. Cognitive status was measured through three dimensions: immediate recall, numerical processing, and visuospatial tasks, with a total possible score of 11. Episodic memory evaluation involved a standardized verbal recall protocol. Interviewers administered a list of 10 unrelated nouns to participants, who were instructed to immediately retrieve as many items as possible. Following a brief delay (5 min), participants repeated the recall task. Each accurate response was allocated 1 point, culminating in a maximum episodic memory score of 20. A composite cognitive score (range: 0–31) was derived by summing both domains. Cognitive impairment was defined as scoring ≥ 1.0 standard deviation (SD) below the mean value of cognitive function assessments, based on prior researches (17, 18).

### Definition of ABSI

ABSI is an anthropometric metric that integrates WC, BMI, and height to quantify body shape and its association with health risks. The calculation method for the modified ABSI formula for Chinese adults, developed by Wang et al. (6), is as follows:


$$\text{ABSI}(kg^{-0.731}m^{1.843})=\frac{\mathrm{WC}(m)}{\mathrm{BMI}{\left(\mathrm{kg}/m^{2}\right)}^{0.731}\times \text{height}{\left(m\right)}^{0.619}}$$

### Covariates

Covariates comprised demographic and health-related variables. Demographic characteristics included age, gender, geographic residence, educational attainment, marital status, and occupational status. Health-related measures consisted of anthropometric parameters (height, weight, BMI, and WC), blood pressure indicators (systolic [SBP] and diastolic [DBP]), medical history (stroke, diabetes, and hypertension), depressive symptoms, social engagement, and lifestyle behaviors (smoking and drinking). BMI was computed using the formula: weight (kg) divided by height squared (m^2^).

Venous blood specimens collected from CHARLS participants were centrifuged, aliquoted, and shipped to the National Center for Disease Control and Prevention (China CDC) in Beijing under standardized protocols. Samples were cryopreserved at −80°C until biochemical analysis at the Clinical Laboratory Center of Capital Medical University (CMU). Enzymatic assays were employed to quantify lipid profiles (total cholesterol [TC], low-density lipoprotein cholesterol [LDL-C], high-density lipoprotein cholesterol [HDL-C] and triglycerides [TG]), while immunoturbidimetry measured high-sensitivity C-reactive protein (hsCRP) concentrations.

Hypertension was defined based on either self-reported physician diagnosis or measured blood pressure thresholds (SBP ≥ 140 mmHg or DBP ≥ 90 mmHg). Diabetes mellitus was diagnosed via self-reported clinical confirmation or laboratory criteria (fasting plasma glucose [FPG] ≥ 126 mg/dL or glycated hemoglobin ≥ 6.5%). Other chronic conditions were ascertained through self-reporting. Depressive risk was evaluated using the 10-item Center for Epidemiologic Studies Depression Scale (CESD-10), with scores ≥ 10 indicating clinically significant symptoms.

Social engagement was assessed through participation in 11 distinct activities during the preceding month: social interactions with peers; recreational group activities (e.g., games, sports); organized exercise/dance sessions; educational/training programs; volunteer/charity work; caregiving for non-cohabitating individuals; financial activities (e.g., stock trading); internet use; community event attendance; assistance to acquaintances; and miscellaneous engagements. Participants reporting involvement in ≥1 activity were classified as socially engaged.

### Statistical analysis

Categorical variables were expressed as frequencies and percentages. Continuous variables were presented as mean ± standard deviation (SD) for normally distributed data, or as median (interquartile range) for skewed distributions. Group comparisons for categorical variables were performed using the chi-square test, while continuous variables were compared using one-way analysis of variance (ANOVA) or the Kruskal-Wallis *H* test, depending on the distribution characteristics. Due to its narrow range, ABSI was multiplied by 100 for scaling purposes. Univariate logistic regression models were used to assess the associations between variables and cognitive impairment. Multivariate logistic regression models were employed to explore the relationship between ABSI and cognitive impairment, with adjustments for potential confounders. Model 1 was unadjusted. Model 2 was adjusted for age and sex. Age, sex, residence, education level, marital status, retirement status, current smoking, current drinking, social engagement, hypertension, diabetes, history of stroke, depressive symptoms, SBP, DBP, FPG, TC, TG, LDL-C, HDL-C, and hsCRP were adjusted in model 3. Additionally, generalized additive models (GAM) with fitted smoothing curves were utilized to investigate the dose–response relationship between ABSI and cognitive impairment. Interaction tests and subgroup analyses were conducted to identify correlations within specific population subgroups. The grouping criteria were as follows: age (45–60 and ≥60 years), gender, place of residence, education level, marital status, retirement status, smoking, drinking, social engagement, as well as the presence of hypertension, diabetes, stroke, and depression. Furthermore, receiver operating characteristic (ROC) curves were constructed to evaluate the Area Under the Curve (AUC) values, and optimal cut-off value of ABSI for the diagnosis of cognitive impairment.

All data analyses were performed using Statistical Product and Service Solutions software (SPSS, version 26.0) and EmpowerStats^2^ (X&Y Solutions, Inc., Boston, MA, USA). A two-tailed *p*-value ≤ 0.05 was considered statistically significant.

## Results

### Clinical characteristics based on ABSI quartiles

The screening process for study participants is illustrated in Figure 1. A total of 6,762 participants were ultimately included in this study. Table 1 presents the baseline characteristics of the participants. Among them, 50.6% were male and 49.4% were female, with a mean age of 58.81 ± 8.91 years. Participants were divided into four groups based on the ABSI quartiles: Q1 (<0.0614), Q2 (0.0614–0.0636), Q3 (0.0637–0.0663), and Q4 (≥0.0664). No significant differences were observed among the ABSI groups in terms of residence, retirement status, stroke prevalence, DBP, or HDL-C levels (*p* > 0.05). Compared to the other three groups (Q1–Q3), the highest ABSI group (Q4) had a higher proportion of individuals aged ≥ 60 years, females, those with education below middle school level, those not married or cohabiting, current smokers, current drinkers, and those with higher social engagement. Additionally, elevated ABSI levels were significantly associated with increased prevalence of hypertension, diabetes mellitus, and depressive symptoms (*p* < 0.05). The higher ABSI group demonstrated significantly elevated levels of SBP, WC, FBP, TC, TG, LDL-C, and hsCRP, along with reduced levels of BMI and height (*p* < 0.05). Furthermore, the higher ABSI group showed significantly lower cognitive performance scores and a higher incidence of cognitive impairment compared to the lower ABSI group (*p* < 0.05).

**Table 1:** The characteristics of participants according to quartiles of ABSI.

		ABSI quartiles			
Characteristics	Q1	Q2	Q3	Q4	*p* value
*N*	1,690	1,691	1,691	1,690	
Age (years)					<0.001
<60	1,258 (74.44%)	1,074 (63.51%)	896 (52.99%)	568 (33.61%)	
≥60	432 (25.56%)	617 (36.49%)	795 (47.01%)	1,122 (66.39%)	
Sex					<0.001
Female	783 (46.33%)	725 (42.87%)	788 (46.60%)	1,042 (61.66%)	
Male	907 (53.67%)	966 (57.13%)	903 (53.40%)	648 (38.34%)	
Residence					0.056
City	607 (35.92%)	615 (36.37%)	674 (39.86%)	611 (36.15%)	
Rural	1,083 (64.08%)	1,076 (63.63%)	1,017 (60.14%)	1,079 (63.85%)	
Education					<0.001
Below middle school	952 (56.33%)	1,041 (61.56%)	1,115 (65.94%)	1,318 (77.99%)	
Middle school and above	738 (43.67%)	650 (38.44%)	576 (34.06%)	372 (22.01%)	
Marital status					<0.001
Others	136 (8.05%)	131 (7.75%)	186 (11.00%)	267 (15.80%)	
Married and cohabiting	1,554 (91.95%)	1,560 (92.25%)	1,505 (89.00%)	1,423 (84.20%)	
Retirement					0.341
No	1,498 (88.64%)	1,496 (88.47%)	1,470 (86.93%)	1,476 (87.34%)	
Yes	192 (11.36%)	195 (11.53%)	221 (13.07%)	214 (12.66%)	
Current smoking					<0.001
No	1,120 (66.27%)	1,098 (64.93%)	1,126 (66.59%)	1,232 (72.90%)	
Yes	570 (33.73%)	593 (35.07%)	565 (33.41%)	458 (27.10%)	
Current drinking					<0.001
No	1,071 (63.37%)	1,026 (60.67%)	1,094 (64.70%)	1,214 (71.83%)	
Yes	619 (36.63%)	665 (39.33%)	597 (35.30%)	476 (28.17%)	
Socializing					<0.001
No	754 (44.62%)	773 (45.71%)	768 (45.42%)	866 (51.24%)	
Yes	936 (55.38%)	918 (54.29%)	923 (54.58%)	824 (48.76%)	
Hypertension					<0.001
No	965 (57.10%)	909 (53.76%)	888 (52.51%)	789 (46.69%)	
Yes	725 (42.90%)	782 (46.24%)	803 (47.49%)	901 (53.31%)	
Diabetes					<0.001
No	1,491 (88.22%)	1,469 (86.87%)	1,402 (82.91%)	1,389 (82.19%)	
Yes	199 (11.78%)	222 (13.13%)	289 (17.09%)	301 (17.81%)	
Stroke					0.149
No	1,662 (98.34%)	1,656 (97.93%)	1,648 (97.46%)	1,644 (97.28%)	
Yes	28 (1.66%)	35 (2.07%)	43 (2.54%)	46 (2.72%)	
Depressive symptoms					<0.001
No	1,127 (66.69%)	1,085 (64.16%)	1,073 (63.45%)	932 (55.15%)	
Yes	563 (33.31%)	606 (35.84%)	618 (36.55%)	758 (44.85%)	
SBP (mmHg)	127.42 ± 20.48	128.21 ± 20.48	129.12 ± 20.78	132.25 ± 22.56	<0.001
DBP (mmHg)	76.06 ± 12.41	75.50 ± 12.37	75.34 ± 11.81	75.10 ± 11.89	0.121
BMI (kg/m^2^)	24.47 ± 4.66	24.05 ± 3.60	23.57 ± 3.41	22.50 ± 3.38	<0.001
Waist (m)	0.81 ± 0.10	0.85 ± 0.10	0.87 ± 0.09	0.89 ± 0.10	<0.001
Height (m)	1.60 ± 0.08	1.60 ± 0.08	1.59 ± 0.08	1.56 ± 0.09	<0.001
FPG (mg/dL)	100.80 (93.42, 110.34)	102.60 (94.68, 112.50)	103.14 (95.58, 115.92)	103.32 (95.22, 115.92)	<0.001
TC (mg/dL)	189.43 ± 37.99	193.38 ± 36.01	195.29 ± 37.79	197.13.35 ± 38.96	<0.001
TG (mg/dL)	100.01 (69.92, 149.57)	106.20 (75.23, 152.22)	107.97 (77.88, 161.07)	109.30 (77.88, 155.98)	<0.001
LDL-C (mg/dL)	113.78 ± 34.09	117.29 ± 33.96	117.29 ± 35.03	118.62 ± 36.19	<0.001
HDL-C (mg/dL)	51.59 ± 14.91	50.80 ± 14.71	50.35 ± 15.46	51.17 ± 15.48	0.104
hsCRP (mg/L)	0.89 (0.49,1.90)	0.93 (0.52,1.85)	1.07 (0.58,2.21)	1.24 (0.65,2.55)	<0.001
Cognitive score	16.64 ± 4.70	15.83 ± 4.68	15.60 ± 4.76	14.15 ± 5.14	<0.001
Cognitive impairment					<0.001
No	1,436 (84.97%)	1,391 (82.26%)	1,363 (80.60%)	1,176 (69.59%)	
Yes	254 (15.03%)	300 (17.74%)	328 (19.40%)	514 (30.41%)	

ABSI, a body shape index; BMI, body mass index; DBP, diastolic blood pressure; FPG, fasting plasma glucose; HDL-C, high-density lipoprotein cholesterol; hsCRP, high-sensitivity C-reactive protein; LDL-C, low-density lipoprotein cholesterol; SBP, systolic blood pressure; TC, total cholesterol; TG, triglyceride.

ABSI was classified as Q1 (<0.0614), Q2 (0.0614–0.0636), Q3 (0.0637–0.0663), and Q4 (≥0.0664).

### Association between clinical characteristics and cognitive impairment

We explored the association between variables and cognitive impairment using univariate logistic regression, as shown in Table 2. Age ≥ 60 years, residing in rural areas, hypertension, depressive symptoms, as well as elevated SBP, HDL-C, and ABSI levels were significantly associated with an increased risk of cognitive impairment (*p* < 0.001). In contrast, male sex, higher education level, being married and cohabiting, retirement, current smoking, current alcohol consumption, engagement in social activities, and elevated BMI and TG levels were associated with a reduced risk of cognitive impairment (*p* < 0.05). However, the following variables showed no significant association with cognitive impairment (*p* > 0.05): diabetes, stroke, DBP, FPG, TC, LDL-C, and hsCRP levels.

**Table 2:** The correlation between clinical characteristics and cognitive impairment.

Characteristics	Statistics	OR (95% CI)	*p* value
Age (years)			
<60	3,796 (56.14%)	Reference	
≥60	2,966 (43.86%)	2.112 (1.874, 2.381)	<0.001
Sex			
Female	3,338 (49.36%)	Reference	
Male	3,424 (50.64%)	0.529 (0.469, 0.597)	<0.001
Residence			
City	2,507 (37.07%)	Reference	
Rural	4,255 (62.93%)	2.009 (1.759, 2.294)	<0.001
Education			
Below middle school	4,426 (65.45%)	Reference	
Middle school and above	2,336 (34.55%)	0.127 (0.104, 0.155)	<0.001
Marital status			
Others	720 (10.65%)	Reference	
Married and cohabiting	6,042 (89.35%)	0.533 (0.449, 0.632)	<0.001
Retirement			
No	5,940 (87.84%)	Reference	
Yes	822 (12.16%)	0.303 (0.234, 0.392)	<0.001
Current smoking			
No	4,576 (67.67%)	Reference	
Yes	2,186 (32.33%)	0.773 (0.681, 0.878)	<0.001
Current drinking			
No	4,405 (65.14%)	Reference	
Yes	2,357 (34.86%)	0.728 (0.643, 0.825)	<0.001
Socializing			
No	3,161 (46.75%)	Reference	
Yes	3,601 (53.25%)	0.531 (0.472, 0.597)	<0.001
Hypertension			
No	3,551 (52.51%)	Reference	
Yes	3,211 (47.49%)	1.293 (1.152, 1.452)	<0.001
Diabetes			
No	5,751 (85.05%)	Reference	
Yes	1,011 (14.95%)	1.028 (0.875, 1.208)	0.736
Stroke			
No	6,610 (97.75%)	Reference	
Yes	152 (2.25%)	1.139 (0.786, 1.650)	0.491
Depressive symptoms			
No	4,217 (62.36%)	Reference	
Yes	2,545 (37.64%)	2.136 (1.900, 2.402)	<0.001
SBP (mmHg)	129.25 ± 21.17	1.010 (1.007, 1.012)	<0.001
DBP (mmHg)	75.50 ± 12.13	0.997 (0.992, 1.002)	0.259
BMI (kg/m^2^)	23.65 ± 3.87	0.936 (0.920, 0.952)	<0.001
FPG (mg/dL)	102.60 (94.68, 113.76)	1.000 (0.998, 1.001)	0.828
TC (mg/dL)	193.81 ± 37.80	1.000 (0.998, 1.002)	0.984
TG (mg/dL)	106.20 (75.23, 154.88)	0.999 (0.998, 1.000)	0.003
LDL-C (mg/dL)	116.75 ± 34.86	0.999 (0.998, 1.001)	0.495
HDL-C (mg/dL)	50.98 ± 15.15	1.010 (1.006, 1.014)	<0.001
hsCRP (mg/L)	1.03 (0.55,2.12)	1.006 (0.999, 1.013)	0.098
100ABSI	6.40 ± 0.46	2.277 (1.993, 2.601)	<0.001

ABSI, a body shape index; BMI, body mass index; CI, confidence interval; DBP, diastolic blood pressure; FPG, fasting plasma glucose; HDL-C, high-density lipoprotein cholesterol; hsCRP, high-sensitivity C-reactive protein; LDL-C, low-density lipoprotein cholesterol; OR, Odds ratio; SBP, systolic blood pressure; TC, total cholesterol; TG, triglyceride.

### Association between ABSI and cognitive impairment

**Figure 2:**
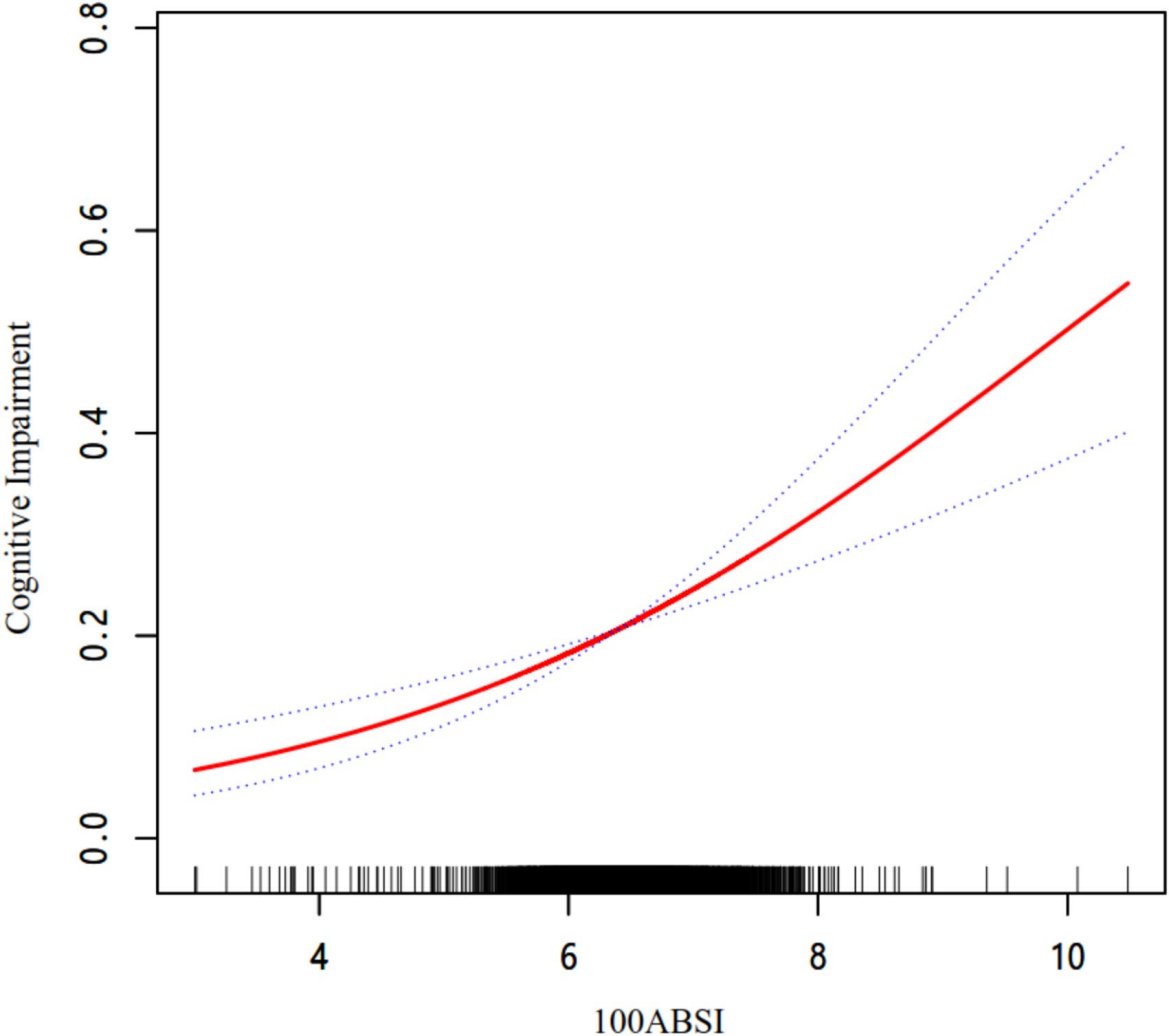
The association between ABSI and cognitive impairment. The relationship was detected after adjusting for age, sex, residence, education, marital status, retirement, current smoking, current drinking, socializing, hypertension, diabetes, stroke, depression symptoms, SBP, DBP, FPG, TC, TG, LDL-C, HDL-C, and hsCRP.

As shown in Table 3, we explored the association between ABSI and cognitive impairment using multivariate logistic regression analysis. As demonstrated in Model 3, after adjusting for all variables, each 0.01-unit increase in ABSI was associated with a 45.7% higher risk of cognitive impairment (odds ratio [OR] = 1.457, 95% confidence interval [CI]: 1.261–1.684; *p* < 0.001). When ABSI was categorized into quartiles, after full adjustment, the risk of cognitive impairment in the Q2, Q3, and Q4 groups increased by 13.1% (OR = 1.131, 95% CI: 0.930–1.378; *p* = 0.218), 14.5% (OR = 1.145, 95% CI: 0.942–1.392; *p* = 0.175), and 48.3% (OR = 1.483, 95% CI: 1.224–1.796; *p* < 0.001), respectively, compared to the Q1 group (*p* for trend < 0.001). As illustrated in Figure 2, the smooth curve fitting revealed a dose–response relationship between ABSI and cognitive impairment, indicating that higher ABSI levels were associated with an increased risk of cognitive impairment.

**Table 3:** Association between ABSI and cognitive impairment.

	OR (95% CI), *p*		
100ABSI	Model 1	Model 2	Model 3
100ABSI	2.277 (1.993, 2.601) < 0.001	1.673 (1.457, 1.919) < 0.001	1.457 (1.261, 1.684) < 0.001
100ABSI quartile (median [range])			
Q1 (5.97 [<6.14])	Reference	Reference	Reference
Q2 (6.26 [6.14–6.36])	1.219 (1.016, 1.464) 0.033	1.155 (0.960, 1.391) 0.127	1.131 (0.930, 1.378) 0.218
Q3 (6.49 [6.37–6.63])	1.361 (1.137, 1.628) 0.001	1.162 (0.966, 1.398) 0.111	1.145 (0.942, 1.392) 0.175
Q4 (6.86 [≥6.64])	2.471 (2.087, 2.926) < 0.001	1.706 (1.426, 2.043) < 0.001	1.483 (1.224, 1.796) < 0.001
*p* for trend	<0.001	<0.001	<0.001

ABSI, a body shape index; CI, confidence interval; OR, Odds ratio.

Model 1 was unadjusted.

Model 2 was adjusted for age and sex.

Model 3 was adjusted for age, sex, residence, education, marital status, retirement, current smoking, current drinking, socializing, hypertension, diabetes, stroke, depression symptoms, SBP, DBP, FPG, TC, TG, LDL-C, HDL-C, and hsCRP.

### Subgroup analyses

As shown in Table 4, no significant interactions were found for all the subgroup variables, including age, sex, education, residence, lifestyle, or comorbidities, indicating a consistent association across these subgroups (*p* for interaction > 0.05).

**Table 4:** The effect size of ABSI on cognitive impairment in the subgroup.

Characteristics	*N*	OR (95% CI), *p* value	*p* for interaction
Age (years)			0.817
<60	3,796	1.423 (1.126, 1.798) 0.003	
≥60	2,966	1.524 (1.265, 1.837) < 0.001	
Sex			0.365
Female	3,338	1.555 (1.297, 1.864) < 0.001	
Male	3,424	1.281 (0.999, 1.643) 0.051	
Residence			0.655
City	2,507	1.524 (1.140, 2.039) 0.004	
Rural	4,255	1.446 (1.222, 1.711) < 0.001	
Education			0.469
Below middle school	4,426	1.435 (1.233, 1.678) < 0.001	
Middle school and above	2,336	1.629 (1.011, 2.625) 0.045	
Marital status			0.186
Married and cohabiting	6,042	1.498 (1.277, 1.758) < 0.001	
Others	720	1.327 (0.931, 1.892) 0.118	
Retirement			0.535
No	5,940	1.435 (1.236, 1.666) < 0.001	
Yes	822	1.998 (1.083, 3.684) 0.027	
Current smoking			0.443
No	4,576	1.490 (1.257, 1.767) < 0.001	
Yes	2,186	1.334 (1.006, 1.770) 0.046	
Current drinking			0.347
No	4,405	1.503 (1.266, 1.783) < 0.001	
Yes	2,357	1.328 (1.011, 1.745) 0.041	
Socializing			0.333
No	3,161	1.344 (1.107, 1.633) 0.003	
Yes	3,601	1.614 (1.296, 2.009) < 0.001	
Hypertension			0.329
No	3,551	1.295 (1.042, 1.609) 0.020	
Yes	3,211	1.600 (1.313, 1.950) < 0.001	
Diabetes			0.398
No	5,751	1.460 (1.248, 1.708) < 0.001	
Yes	1,011	1.390 (0.944, 2.047) 0.096	
Stroke			0.736
No	6,610	1.450 (1.252, 1.679) < 0.001	
Yes	152	1.373 (0.521, 3.618) 0.522	
Depressive symptoms			0.104
No	4,217	1.615 (1.306, 1.996) < 0.001	
Yes	2,545	1.346 (1.102, 1.643) 0.004	

CI, confidence interval; OR, Odds ratio; BMI, body mass index.

Note 1: Above models were adjusted for age, sex, residence, education, marital status, retirement, current smoking, current drinking, socializing, hypertension, diabetes, stroke, depression symptoms, SBP, DBP, FPG, TC, TG, LDL-C, HDL-C, and hsCRP.

Note 2: In each case, the model was not adjusted for the stratification variable.

Note 3: The ABSI value was multiplied by 100 for scaling.

### ROC curves

**Figure 3:**
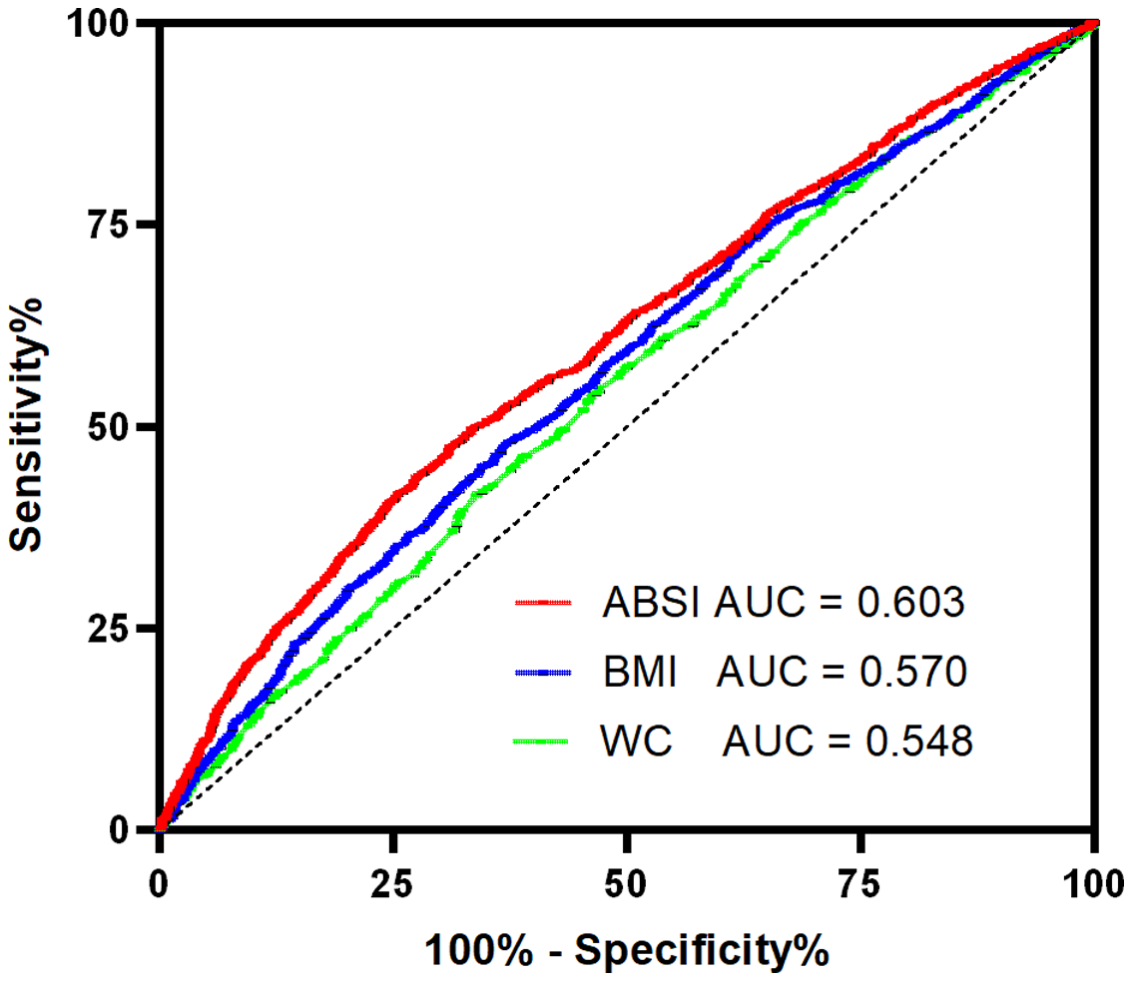
ROC curves of ABSI, BMI and WC with reference line for cognitive impairment. ABSI, a body shape index; AUC, area under the curve; BMI, body mass index; ROC, receiver operating characteristic; WC, waist circumference.

Figure 3 presented a ROC curve analysis comparing the diagnostic performance of three different metrics: ABSI (AUC = 0.603), BMI (AUC = 0.570), and WC (AUC = 0.548). The curve illustrated the trade-off between sensitivity and specificity, with ABSI showing the highest AUC, indicating better diagnostic accuracy compared to BMI and WC. In this study, the cut-off value for ABSI was determined to be 0.0652, demonstrating a sensitivity of 0.475 and a specificity of 0.690.

## Discussion

This study revealed a significant positive relationship between elevated ABSI levels and an increased risk of cognitive impairment in Chinese middle-aged and older adults. The dose–response relationship observed in this study suggested that higher ABSI levels was progressively associated with a greater likelihood of cognitive decline. These results highlight the potential utility of ABSI as a novel anthropometric indicator for identifying individuals at risk of cognitive impairment.

The relationship between BMI and cognitive impairment/dementia is complex and controversial. Underweight (BMI < 18.5 kg/m^2^) was consistently associated with increased cognitive impairment risk (19–22), while midlife overweight or obesity increased dementia risk and late-life overweight/obesity may protect against cognitive decline (23, 24). Significant BMI changes (≥5%) or midlife weight increase followed by decline were linked to faster cognitive decline (25, 26). A study including a total of 1.35 million individuals, revealed that elevated BMI was associated with adverse outcomes over extended follow-up durations, whereas reverse causation effects may lead to an apparent protective association in studies with limited follow-up time (27). However, a longitudinal study spanning 32 years revealed no significant correlation between elevated or reduced midlife body weight or BMI and the incidence of late-onset dementia (28). These debates are, in part, associated with the obesity paradox or reverse epidemiology. A lean body composition did not invariably signify optimal health, and potential selection and treatment biases may foster the development of an obese yet comparatively healthier phenotype (29–31). Furthermore, these issues underscore the inherent limitations of BMI, such as its inability to differentiate fat mass from muscle mass and its inadequacy in evaluating abdominal obesity. BMI often inaccurately represented the health status of older populations, resulting in a significant underestimation of obesity’s contribution to morbidity and mortality risks (32).

ABSI, which integrates WC, BMI, and height, provides a more nuanced assessment of body shape and its associated health risks. The ABSI formulas derived for different populations exhibit distinct BMI and height exponents. Krakauer et al. (5) initially developed the US adult ABSI formula (BMI exponent: 0.681; height: 0.548). Subsequent studies reported variations: Cheung’s Indonesian formula (0.632 and 0.463) (33) and Chung’s Korean formula (0.726 and 0.390) (34). Wang et al.’s Chinese ABSI differs significantly, underscoring the necessity of population-specific formulas for accurate abdominal obesity assessment (6). Previous studies show inconsistent ABSI-disease associations: weaker diabetes/hypertension links in Chinese (35, 36) and Japanese (37) populations but strong diabetes prediction in Spanish obese individuals (38). These discrepancies may stem from using Krakauer’s original formula (5), highlighting the importance of employing population- and gender-stratified ABSI formulas for reliable chronic disease risk evaluation.

Obesity contributes to cognitive decline through a variety of complex physiological and pathological mechanisms. It induces chronic systemic low-grade inflammation, particularly in adipose tissue, which secretes a wide array of pro-inflammatory cytokines such as tumor necrosis factor-alpha (TNF-α), tumor necrosis factor-beta (TNF-β), interleukin-1 beta (IL-1β), IL-6, IL-12, and IL-8. This inflammatory state, along with leptin resistance and insulin resistance, disrupts insulin signaling pathways, triggering neuroinflammation. Consequently, this leads to neurotrophic deficiency, neuronal apoptosis, synaptic damage, and an imbalance in brain homeostasis, ultimately resulting in cognitive dysfunction (39–42). The elevated levels of hsCRP and lipid profiles observed in the higher ABSI group in our study support the role of systemic inflammation and metabolic disturbances in this association. Furthermore, dysfunction in adipose tissue among diabetic individuals can cause adipose-derived extracellular vesicles (EVs) to cross the blood–brain barrier (BBB). These EVs can induce synaptic damage and cognitive decline through microRNA (miRNA) signaling pathways (43). Additionally, obesity-related gut microbiota dysbiosis may exacerbate BBB permeability. Certain bacteria that secrete amyloid proteins or lipopolysaccharides can upregulate pro-inflammatory factors via the gut-brain axis or BBB, further impairing cognitive function (41, 44). Moreover, obesity adversely affects brain plasticity and structure, correlating with reduced brain volume, cortical atrophy, and decreased gray and white matter volumes (45, 46).

In our study, subgroup analyses revealed that elevated ABSI increased the risk of cognitive dysfunction in both the low- and high-education groups, though the odds ratio (OR) was slightly higher in the high-education group. Previous studies have suggested that formal education may help maintain cognitive function in middle and late life while delaying symptom progression. Increased years of education can establish “cognitive reserve,” improve socioeconomic status (SES), and reduce dementia risk (1). However, higher education does not always translate into stronger or more extensive social connections, and social support may even decline with advanced education (47). Currently, no studies have evaluated the magnitude of ABSI’s impact on cognition across different educational strata. We hypothesize that the differential effects of ABSI across education groups may be attributed to three interrelated mechanisms: First, while higher-educated individuals generally benefit from greater health literacy and resource access (theoretically buffering metabolic risks), their elevated OR suggests that health-seeking behaviors (e.g., earlier clinical detection) may paradoxically amplify the recorded impact of ABSI-related biological damage through detection bias. Second, chronic exposure to socioeconomic adversities (e.g., poverty, chronic stress) in low-education populations could induce metabolic adaptations that attenuate ABSI-related risk expression. In contrast, ABSI elevation in high-education individuals likely signifies a breakdown of such compensatory mechanisms, leading to disproportionately steeper risk increments. Third, the underrepresentation of highly educated individuals in high-ABSI strata implies education’s intrinsic protective role; however, when ABSI rises despite high education, it may signal distinct etiological pathways (e.g., genetic predisposition or unmeasured environmental triggers), thereby magnifying the observed OR. These speculative mechanisms warrant validation in studies with detailed phenotyping and longitudinal designs.

The ROC curve analysis demonstrated that ABSI had superior diagnostic accuracy for cognitive impairment compared to BMI and WC, as evidenced by its higher AUC. The optimal ABSI cut-off value of 0.0652 identified in this study provides a practical threshold for risk stratification, although further validation in independent cohorts is warranted. Current evidence regarding the association between ABSI and cognitive function remains limited. A study conducted in the U.S. population reported an AUC of 0.530 for ABSI, slightly superior to BMI (0.505) and WC (0.496) (14). Consistent with our findings, although ABSI demonstrates modest predictive power, it consistently outperforms BMI and WC in predicting cognitive impairment. While the incremental value of ABSI may not currently justify replacing BMI and WC entirely, it could serve as a complementary tool to traditional anthropometric measures for population-level cognitive risk assessment.

This study has several strengths, including its large sample size, nationally representative design, and comprehensive adjustment for potential confounders. This marks the first application of the modified ABSI, tailored for Chinese adults, in assessing cognitive risk. However, certain limitations should be acknowledged. First, the cross-sectional design precluded the establishment of causal relationships between ABSI and cognitive impairment. Longitudinal studies are needed to confirm the temporal association and explore potential mechanisms. Second, cognitive function was assessed using a composite score rather than a clinical diagnosis of dementia or mild cognitive impairment, which may limit the generalizability of the findings. Third, due to database constraints and data completeness considerations, variables such as physical activity, diet, and APOE ε4 status could not be included in our analyses. Our assessment of psychoactive substances was limited to current smoking and drinking status. Detailed parameters including dosage, duration of use, and dependence criteria were not available. Fourth, while ABSI provides a more refined measure of body shape, it does not account for other factors such as muscle mass or fat distribution, which may also influence cognitive health. Finally, the study population was limited to Chinese adults, and further research is needed to determine whether these findings are applicable to other ethnic groups.

## Conclusion

This study provides compelling evidence that higher ABSI levels are associated with an increased risk of cognitive impairment in middle-aged and older Chinese adults. The findings highlight the potential utility of ABSI as a simple and effective tool for identifying individuals at risk of cognitive decline. Future research should focus on elucidating the underlying mechanisms and exploring the potential benefits of interventions targeting central obesity and metabolic health for cognitive preservation. Public health strategies aimed at reducing the burden of cognitive impairment should consider incorporating ABSI as part of routine health assessments, alongside traditional measures such as BMI and WC.

## Data Availability

The datasets presented in this study can be found in online repositories. The names of the repository/repositories and accession number(s) can be found in the article/supplementary material.

## Footnotes

^1^ http://charls.pku.edu.cn

^2^ http://www.empowerstats.com
